# System Matrix Analysis for Computed Tomography Imaging

**DOI:** 10.1371/journal.pone.0143202

**Published:** 2015-11-17

**Authors:** Liubov Flores, Vicent Vidal, Gumersindo Verdú

**Affiliations:** 1 Departamento de Sistemas Informáticos y Computación, Universitat Politècnica de València, Valencia, Spain; 2 Departamento de Ingeniería Química y Nuclear, Universitat Politècnica de València, Valencia, Spain; Chongqing University, CHINA

## Abstract

In practical applications of computed tomography imaging (CT), it is often the case that the set of projection data is incomplete owing to the physical conditions of the data acquisition process. On the other hand, the high radiation dose imposed on patients is also undesired. These issues demand that high quality CT images can be reconstructed from limited projection data. For this reason, iterative methods of image reconstruction have become a topic of increased research interest. Several algorithms have been proposed for few-view CT. We consider that the accurate solution of the reconstruction problem also depends on the system matrix that simulates the scanning process. In this work, we analyze the application of the Siddon method to generate elements of the matrix and we present results based on real projection data.

## Introduction

In medicine, the diagnosis based on computed tomography (CT) is fundamental for the detection of abnormal tissues that are frequently not clearly distinguished by radiologists. However, excessive X-ray radiation exposure is not desirable. In the last three decades, several CT imaging methods have been proposed to obtain the internal structure of an object; for example, [1].

In CT, owing to the physical conditions of the data acquisition process, it is common to find noisy, incomplete set of unequally spaced projections. In these cases, iterative methods demonstrated their superiority in reconstruction of images compared to analytical methods. They are capable to provide the optimal reconstruction of the image from a limited set of projections [2].

However, for practical use, iterative algorithms must be as efficient as possible. One way to reduce the radiation dose is to decrease the number of rotations during data acquisition. As a consequence, undesired artifacts appear in the reconstruction. With the development of compressed sensing theory [3, 4], compressed-sensing-based iterative algorithms have drawn much attention in medical imaging. Subsequently, many algorithms have been developed for few-view CT image reconstruction. Yu and Wang [5] adapted a soft-threshold filtering (STF) algorithm for total variation (TV) minimization in image reconstruction. With the aim to eliminate the streak artifacts and preserve the edge structure, Yu and Zeng [6] developed an iterative reconstruction algorithm based on weighted total difference (WTD) minimization for few-view CT. To solve the model effectively, the soft-threshold filtering method and a fast iterative shrinkage thresholding algorithm have been employed to accelerate the convergence speed.

To update the current reconstruction, both methods, STF for TV and WTD, use the simultaneous algebraic reconstruction technique (SART) which is a classical reconstruction algorithm in CT imaging [7]. However, owing to the lineal convergence of this method, the computational cost of the algorithm is high, especially in 3D reconstruction, which makes SART difficult for practical uses.

In our previous research, we proposed the parallel implementation of the method for sparse linear equations and sparse least squares (LSQR) to resolve the reconstruction problem [8, 9]. In this work, we analyse the system matrix that simulates the scanning process and affects the quality of the reconstructed image. Furthermore, we apply the Siddon method to generate the elements of the system matrix and present results based on real projection data.

The rest of the paper is organized as follows: in the next section, the mathematical aspects of the methods used in this work are presented. Subsequently, we describe the methodology used to perform the experiments and present some results of the implementation of these algorithms. Finally, we summarize our conclusions.

## Mathematical Aspects

The problem of image reconstruction from projections can be can considered as a system of linear equations of the form:

Ax≈b, (1)

where the system matrix *A* simulates CT operation and may not be square; its elements depend on the projection number and the angle at which the projections have been acquired. The values of the column matrix ***x*** represent the intensities of the image, and the column matrix ***b*** represents projections collected by a scanner.

For a given angle, we assume that the number of projections ranges from 1 to *m*. For *k* different angles, in Eq (1), ***b*** has *M = mxk* elements, ***x*** has *N* elements, and ***A*** is an *MxN* rectangular matrix.

$$A=\left[\begin{array}{c} \begin{array}{cc} a_{11}\;\;\;\;a_{12}\;\;\;\ldots & a_{1N}\\ a_{21}\;\;\;\;a_{22}\;\;\;\ldots & a_{2N} \end{array}\\ \ldots \ldots \ldots \ldots \ldots \ldots \ldots \ldots \\ a_{M1}\;\;\;\;\;a_{M2}\;\;\ldots \;\;\;a_{MN} \end{array}\right],\;\;\;\;\;\;\;b=\;{\left[b_{1},\;b_{2}\ldots \;b_{M}\right]}^{T},\;\;\;\;\;\;\;\;x={\left[x_{1},\;x_{2}\ldots \;x_{N}\;\right]}^{T}.$$

### 2.1 Analysis of the system matrix

Many properties of the reconstructed image depend on the approximations when calculating the system matrix. In this work, we intend to find the best way to compute elements of the matrix on a rectangular grid. It was shown that among methods such as Joseph, Siddon or the cube method, the Siddon method gives the best results in calculating the elements of the system matrix *A* [10].

#### 2.1.1 Description of the matrix

**Fig 1:**
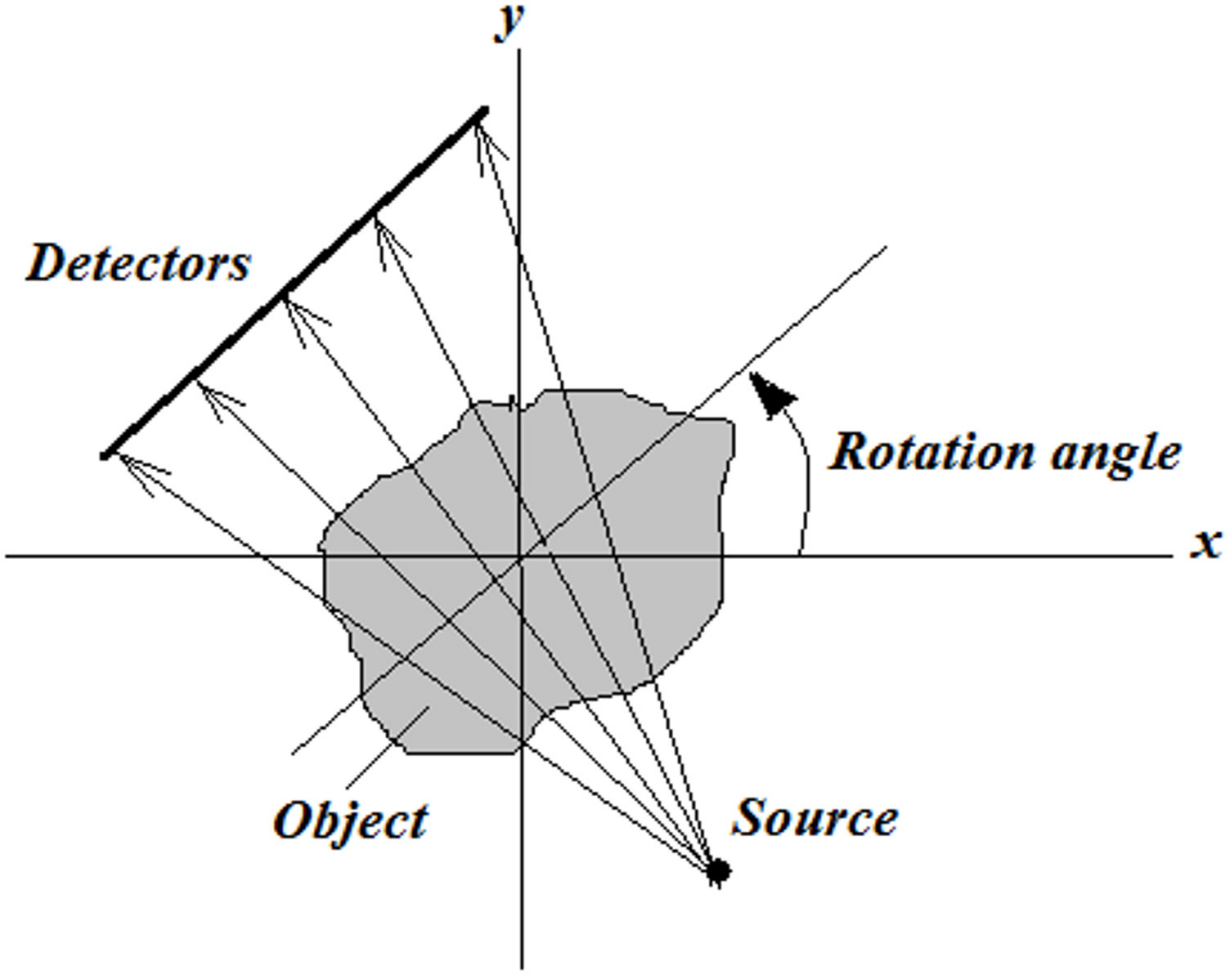
The data acquisition process during scanning.

**Fig 2:**
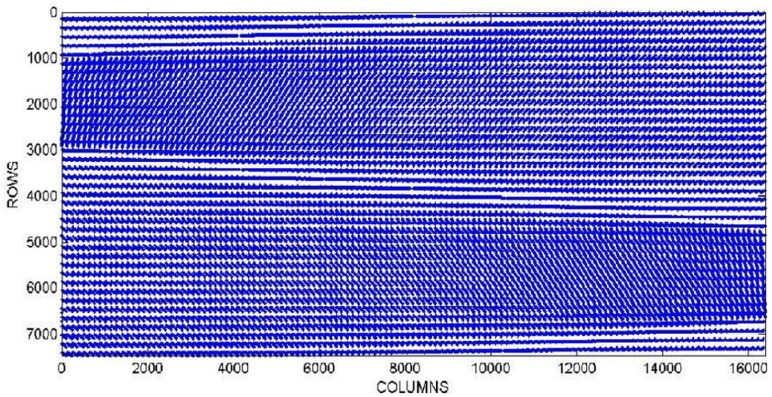
Sparsity pattern of the system matrix. The figure shows a part of the matrix data structure that corresponds to 7000 rows and 16000 columns. doi:10.1016/j.procs.2013.05.308.

In CT imaging, during the dada acquisition, the scanning range is 0−360 degrees (see Fig 1). As described in details previously [8], in this range, the system matrix that simulates the data acquisition process contains symmetric blocks of data structure as shown in Fig 2.

In practice, *A* is a rectangular nonsymmetrical sparse matrix and therefore it is appropriate to use a compact storage format that only allows the storage of nonzero elements, such as compact sparse row (CSR) or compact sparse column (CSC). The dimensions of *A* increase proportionally to the resolution of the image to be reconstructed and the number of projections, thus increasing the computational cost.

#### 2.1.2 Simulation of elements

**Fig 3:**
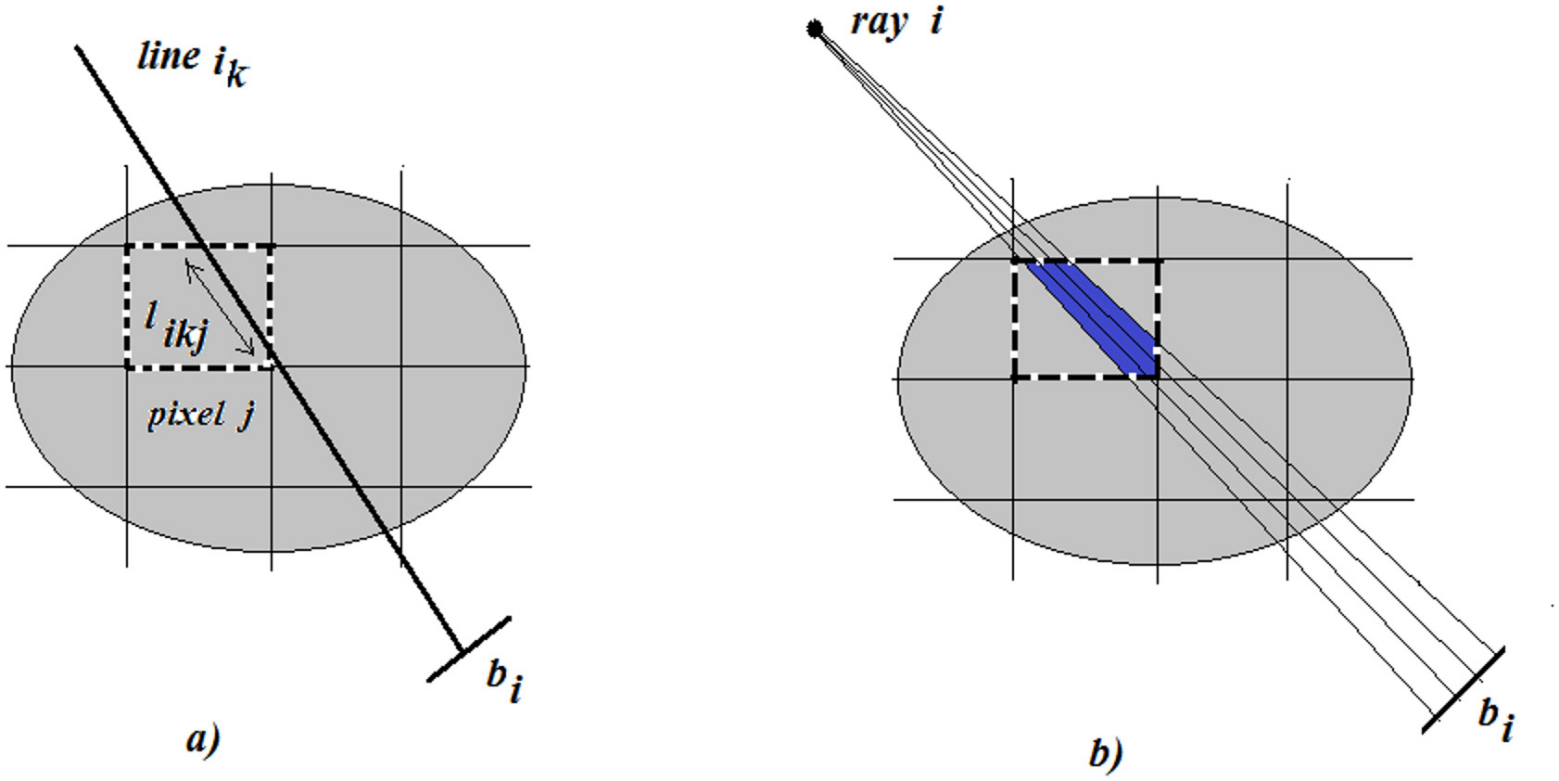
Schematic representation of an X-ray passing through pixel *j* and registered by a detector *b*_*i*_.

The elements of the matrix give the proportion of ray *i* passing through the pixel *j* (Fig 3(a)). One ray can be simulated with a set of lines that traverse the digital image and are registered by a detector on the other side. The crossed area of the pixel gives the corresponding element of the system matrix. The simulation is shown in Fig 3(b).

An element *a*_*ij*_ of matrix *A* can be expressed by the equation:

$$a_{ij}=\;\overset{n}{\underset{k=1}{\sum }}l_{ikj},$$ (2)

where *n* is the number of lines per ray.

**Fig 4:**
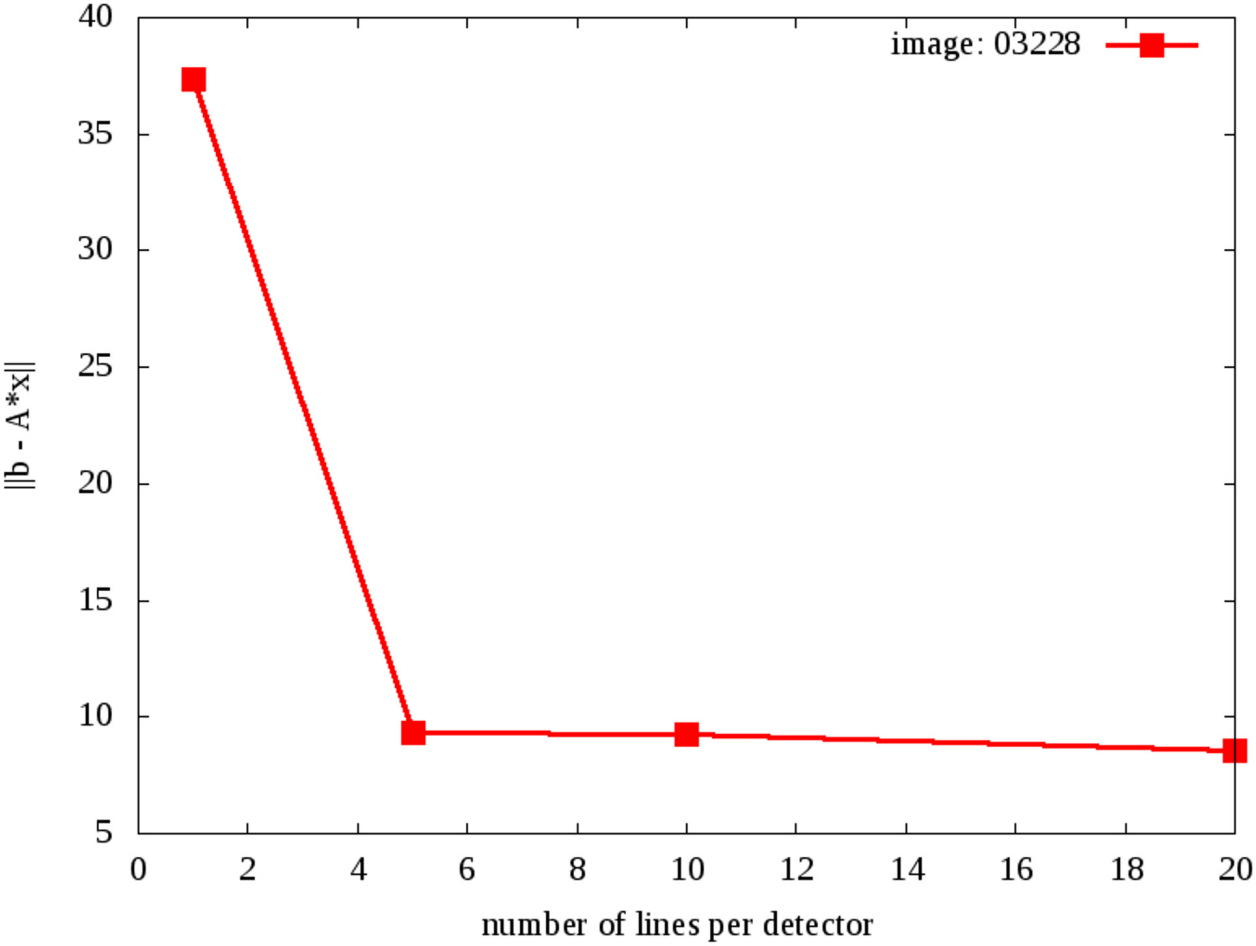
Norm error as a function of number of lines per detector after 100 iterations.

How many lines should be taken to simulate one ray? We have performed simulations with different number of rays and analyzed the norm of the error of the reconstructed image. In our simulations, the length of the detector panel was 500 mm and we considered 256 detectors. Therefore, the length of each detector was 1.9 mm. Fig 4 shows the behavior of the norm of the error ||*b–Ax*|| as a function of number of lines per detector. We concluded that the error decreases and becomes practically stable when using 20 lines per detector, or 5 lines per 1 mm of the detector.

This conclusion has been used in our simulations when we generated the system matrix with the Siddon method [11].

#### 2.1.3 Using the symmetry of the block structure

**Fig 5:**
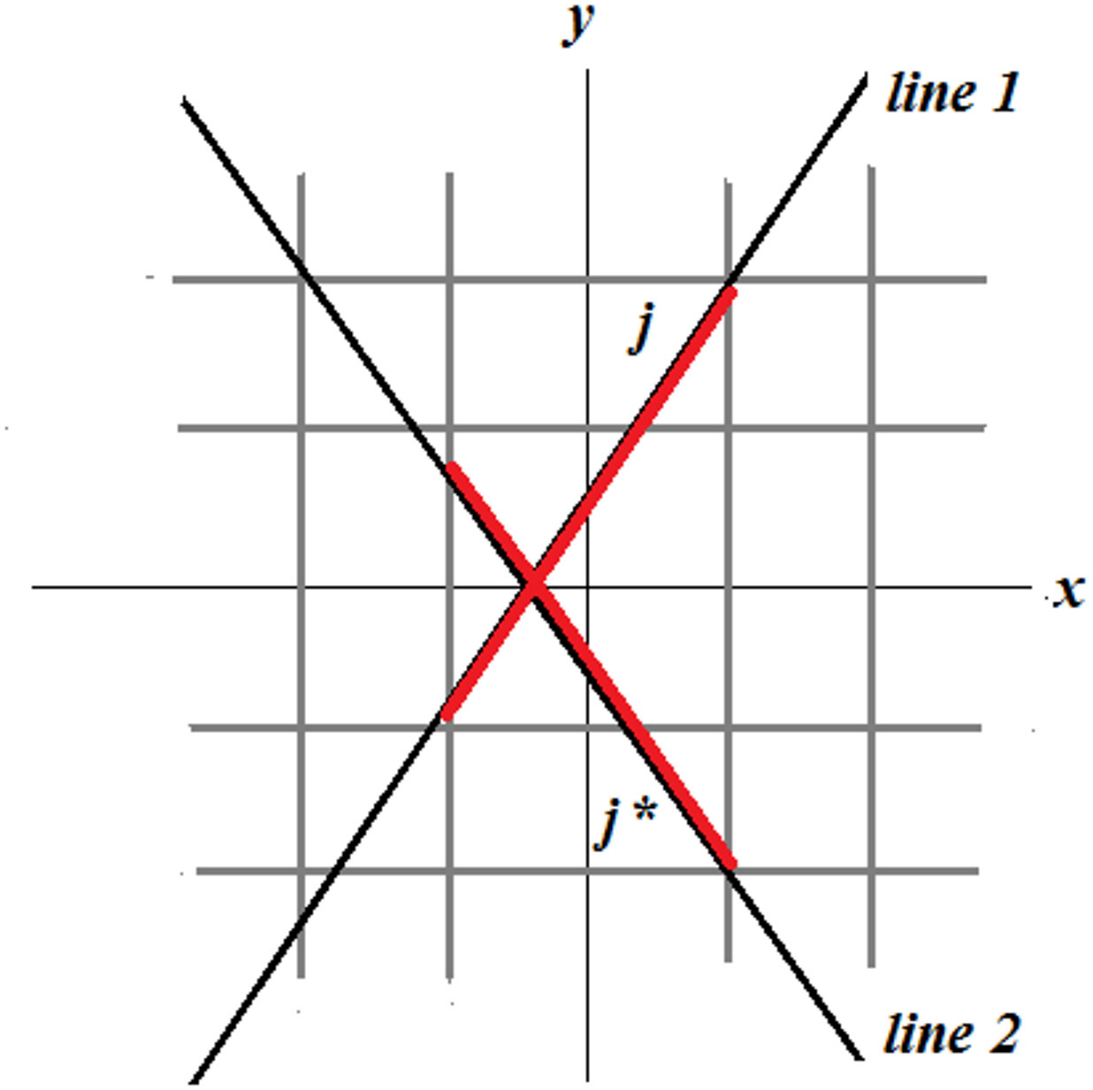
The symmetry of the block data structure of the system matrix allows to reuse the matrix generated in the range 0–90 degrees.

The symmetric block structure of the matrix *A* allows us to generate the matrix only in the range 0–90 degrees and project the data in the range 0–180 or 0–360 degrees. For example, in Fig 5, line 1 and line 2 are symmetric with respect to the *x* axis and the relation between the pixels crossed by these two lines can be expressed as follows:

Let *x =* {*x*_*1*_, *x*_*2*_ *… x*_*N*_} be a digital image with *N = n*x*n*.

For pixel *j*:

*row = int (j*,*n)* denotes the integer division that returns the entire part after division of *j* by *n*.

*column = mod (j*,*n)* represents the modulo operation that determines the reminder after the division of *j* by *n*.

Corresponding pixel j* is: j* = (n–row) n + column.

The symmetric block structure of the matrix *A* allows us to reduce system memory usage, loading and generation time of the matrix.

### 2.2 The Siddon method

The projection of a ray is proportional to the sum of projections of the lines that compose the ray. The projection of each line on a pixel is similated by the length of the intersection of this line and the pixel, weighted by the density of this pixel.

In 1985, Siddon proposed a method to calculate the length of a ray that crosses the rectangular grid [11] that represents a digitalized image. The algorithm can be summarized as follows:

1. Compute all intersection points of the line *i* with the grid limits.
2. Order the set of intersection points.
3. Identify the pixel *j*.
4. Calculate the length between the intersection points that define the pixel *j*.

**Fig 6:**
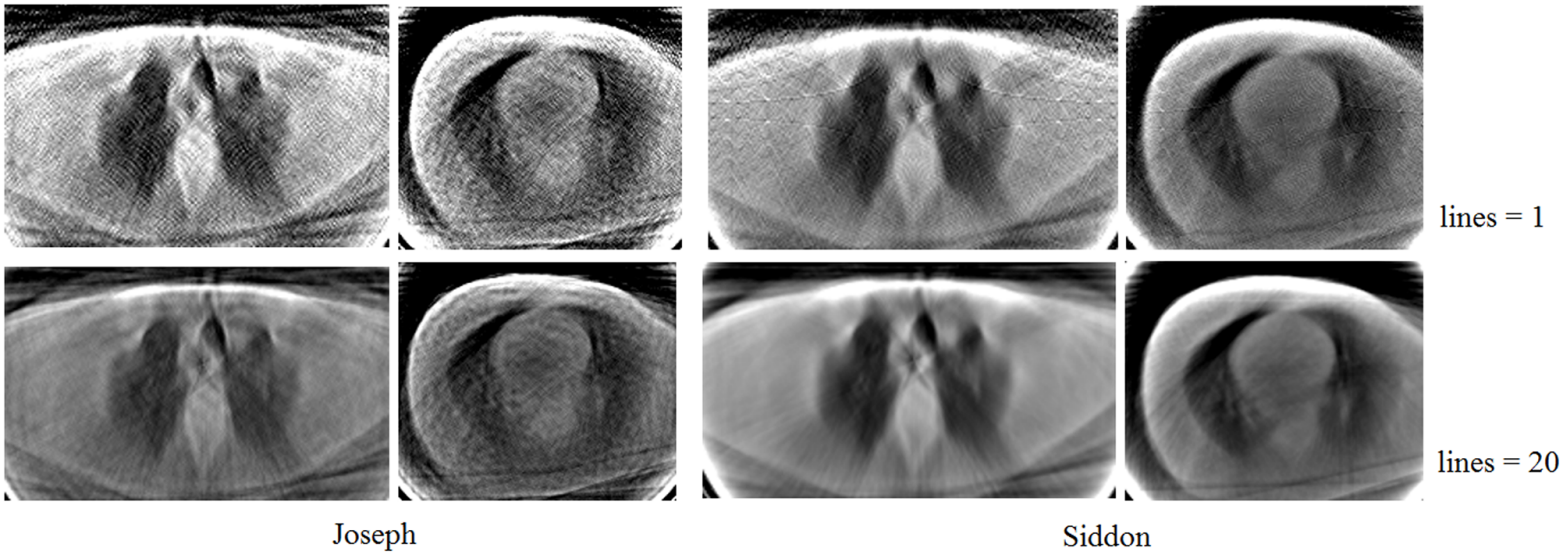
Reconstructions based on matrices generated with Joseph’s and Siddon’s methods.

In [10] it is shown that the Siddon’s method gives better results in calculating elements of the system matrix. For comparison purposes, we generated the matrices with Siddon’s and Joseph’s [12] methods simulating the scanning process with 256 detectors and considering 1 and 20 lines per detector. The source-detector distance was 140 cm, the detector panel length was 50 cm, and the maximum opening angle of the beam was 20.25 degrees. Fig 6 shows reconstructions of 256x256 pixels made with the LSQR algorithm [13] from 100 projections using matrices generated with Siddon’s and Joseph’s methods. To evaluate the reconstructions we used measures described in seccion 2.4. The results of quantitative comparison of the reconstructions with the reference image reconstructed from 400 projections are summarized in Table 1. The results confirm that the Siddon’s approach provides better quality reconstructions.

**Table 1:** Quality comparison of reconstructions based on matrices generated with Joseph’s and Siddon’s methods.

Measure	Joseph	Siddon
MSE	0.1250	0.0820
PSNR	57.1617	58.9929

In this work, we have adopted the Siddon’s approach to compute the elements of the matrix *A* in a rectangular grid considering 5 lines per mm per detector.

### 2.3 Reconstruction method

The problem of image reconstruction from a limited number of projections, described by Eq 1, is considered as an ill-posed problem. To determine the solution, we adopted the LSQR method [13] that solves Eq 1 by minimizing the norm: min ||*Ax–b*||_2_. The LSQR method is based on the bidiagonalization procedure of Golub and Kahan [14] and it is the most reliable algorithm when *A* is ill-conditioned. The LSQR technique generates a sequence of approximations {*x*_*k*_} such that the residual norm ||*r*_*k*_||_2_, where *r*_*k*_ *= b—Ax*_*k*_, decreases monotonically. The matrix *A* is normally large and sparse and is used only to compute products of the form *Av* and *A* ^*T*^ *u* for various vectors ***v*** and ***u***. The main steps of the LSQR procedure are described in [13].

### 2.4 Performance evaluation

To perform a statistical analysis of the quality difference between the reference image (*I*_*1*_) and a reconstructed (*I*_*2*_) one, we used the following functions:

- Mean square error:

$$MSE=\overset{n}{\underset{i=1}{\sum }}\overset{n}{\underset{j=1}{\sum }}{\left[I_{1}\left(i,j\right)-\;I_{2}\left(i,j\right)\right]}^{2},$$

- Peak signal-to-noise ratio:

$$PSNR=\frac{1}{n^{2}}log_{10}\frac{MAX_{I}^{2}}{MSE},$$

where *n* corresponds to the resolution (*n*x*n* pixels) of the reconstructed image and *MAX*_*I*_ is the maximum possible pixel value of the image.

## Results and Discussion

In this work we used projection data acquired from a CT scanner of the Hospital Clínic Universitari of València, which is a CT simulator of Metaserto model with an attached Kermath tomography system. The system has 512 detectors situated along the detector panel of 50 cm. The distance between the radiation source and the detectors is 1.4 m. The maximum opening angle of the beam is 20.25 degrees. One measure of such system consists of 200 rotations in π radians.

So, the initial set of the experimental data (200 projections) were collected by a scanner with 512 sensors in the range 0–180 degrees with a step of 0.9 degrees. We extended the given set up to 360 degrees using the symmetry of the scanning process. We aimed to analyze the capacity of the LSQR algorithm in a parallel reconstruction of images from a smaller number of projections. For this purpose, two sets of equally spaced projections (with an angle step of 3.6 degrees) were derived from the initial set: the first in the range 0–360 degrees (100 projections) and the second in the range 0–180 degrees (50 projections). These projections were used to reconstruct images in the ranges 0–360 and 0–180 degrees with LSQR and the corresponding matrices generated with the Siddon method.

The results have been obtained on the system gpu.dsic.upv.es with a CPU of 2.6 GHz and NVIDIA TESLA K20c graphical prosessing unit (GPU) card that belongs to the Departamento de Sistemas Informáticos y Computación of the Universistat Politècnica de València. One GPU card has been used to perform the experiment. Such a GPU card has a total number of 2496 CUDA cores with 5 GB memory, shared by all processor cores. The algorithm was implemented using the CUDA programming mode.

**Fig 7:**
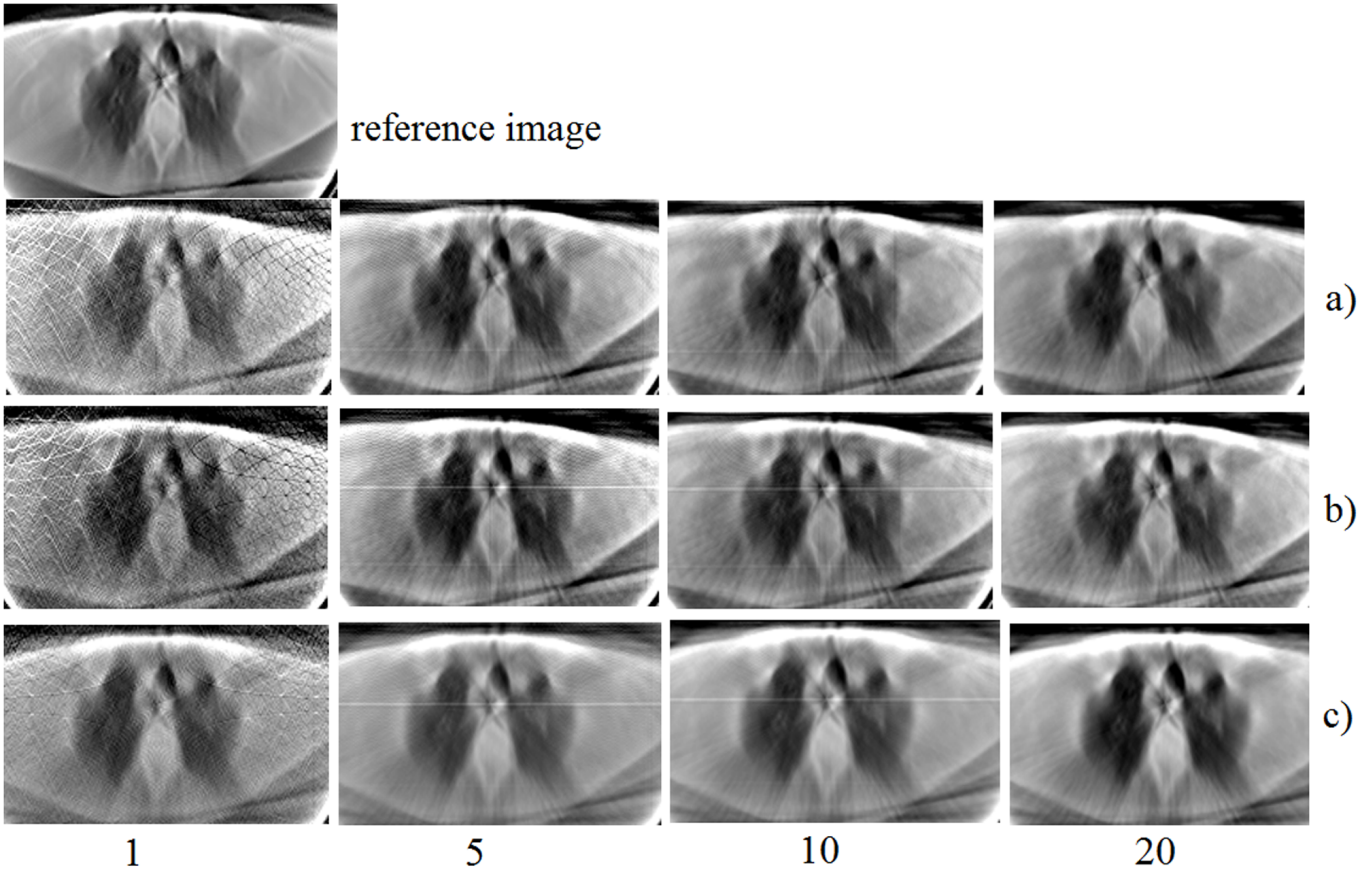
Reconstructed images after 100 iterations using matrices simulated in ranges: 0–90 (a), 0–180 (b), and 0–360 (c) degrees and considering 1, 5, 10, and 20 lines per detector. The reference image is reconstructed using the FBP algorithm from the complete set of projections (200).

In the experiment, the system matrices have been generated with the Siddon method in the ranges 0–90, 0–180 and 0–360 degrees using different number of lines per detector. The images were reconstructed from 50 projections in the range 0–180 degrees and 100 projections in the range 0–360 degrees. Fig 7 shows the resulting reconstructions using such matrices and the LSQR algorithm.

We can see that the image was successfully reconstructed with the matrix generated in the range 0–90 degrees, which allows the reduction of system memory and matrix generation time, as shown in Table 2. Furthermore, the loading time of the input data is also reduced. Consequently, the reconstruction time (including loading time) of a slice of 256x256 pixels with the matrix generated in the range 0–90 degrees is almost four times faster than the obtained using the matrix in the range 0–360 degrees.

**Table 2:** Memory size, generation, and reconstruction time used by different system matrices.

Matrix range	Size	Generation time	Reconstruction time
0–360 degrees	154 MB	1379 s	2.25 ms
0–180 degrees	77 MB	680 s	1.13 ms
0–90 degrees	39 MB	340 s	0.68 ms

Comparison of results in the ranges 0–90 and 0–180 degrees demonstrates that it is more efficient to generate the system matrix in the range 0–90 degrees, complete it to 0–180 o 0–360 degrees and then reconstruct the image, than to use a pre-computed matrix in the range 0–180 o 0–360degrees from the beginning.

**Fig 8:**
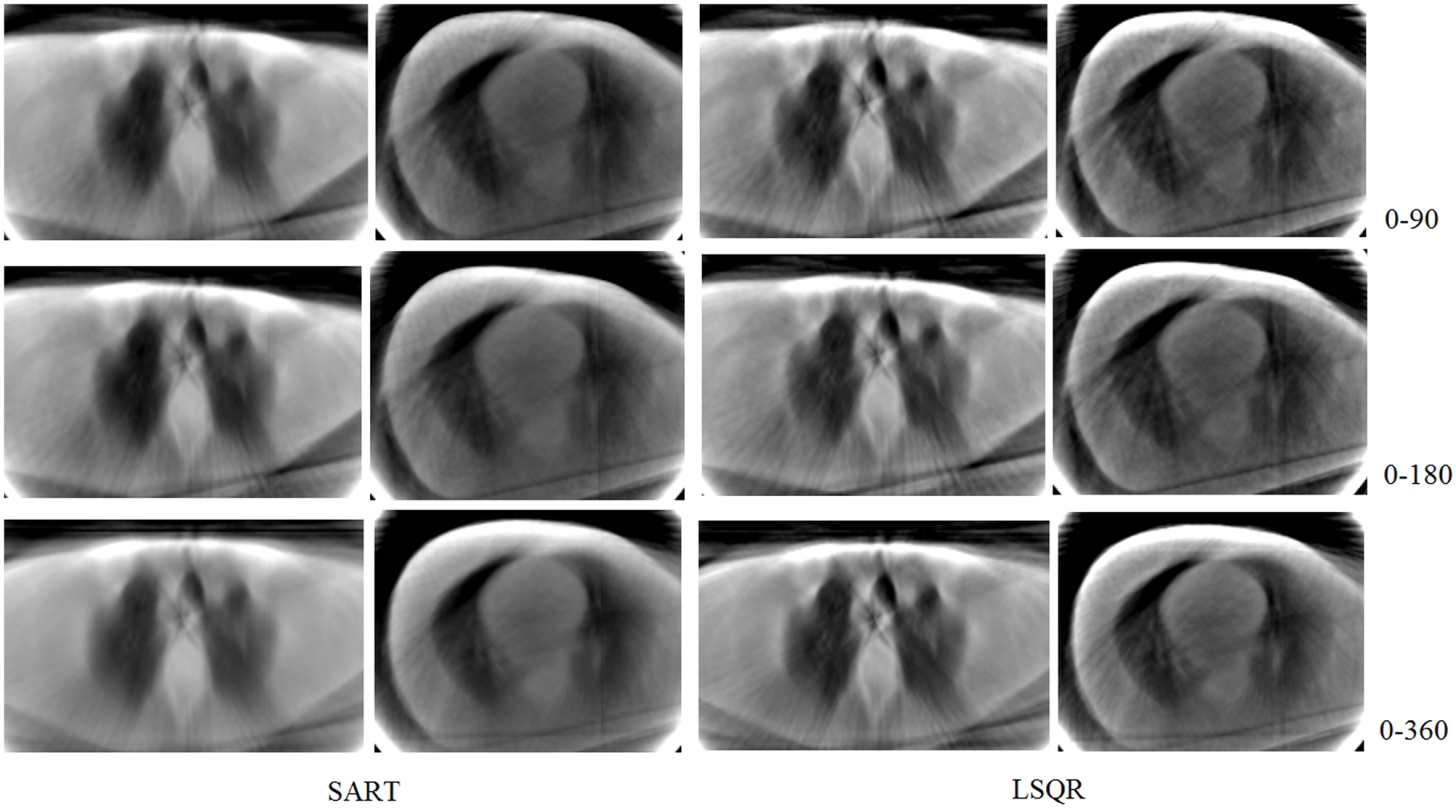
Images reconstructed with SART and LSQR methods using matrices simulated in ranges: 0–90, 0–180, and 0–360 degrees and considering 20 lines per detector. The reconstructions are presented after 100 iterations when convergence is reached.

The experiment has been repeated using the simultaneous algebraic reconstruction technique (SART), which is a classical iterative reconstruction algorithm in CT imaging. The iteration formula in SART is taken as in the reference [6]. Fig 8 shows the resulting reconstructions after 100 iterations. It is observed that the images made by SART and LSQR are very similar. However, the computational cost per iteration for the slice of 256x256 pixels reconstructed with the matrix generated in the range 0–360 degrees is 12.6 milliseconds for SART and 2.3 milliseconds for LSQR. In addition, these methods lead to different image qualities. The peak signal-to-noise ratio (PSNR) is for SART 56.51 points and for LSQR 58.99 points.

**Fig 9:**
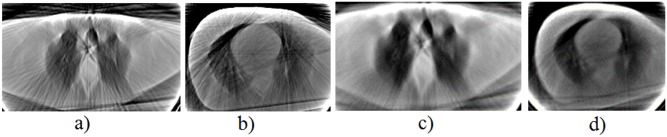
Images reconstructed with FBP (a-b) and LSQR (c-d) methods.

We also compare the LSQR method with the classical filtered back projection (FBP) method [1]. In Fig 9, the results obtained from 50 projections show that LSQR reconstructions present less artifacts and prove that iterative methods are more suitable for reconstruction from less number of projections.

Fig 7 shows that the quality of the images improves as the number of lines per detector increases. Furthermore, the difference becomes less prominent when this number is between 10 and 20. This agrees with Fig 4, which shows the error as a function of the number of lines.

**Fig 10:**
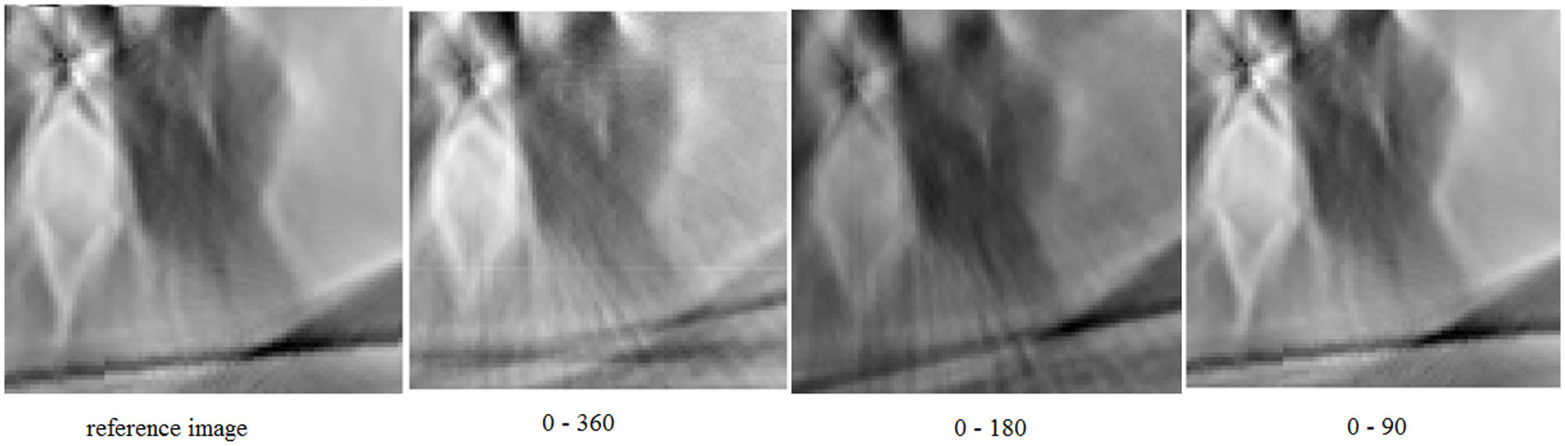
Magnified view of the images reconstructed with LSQR using matrices generated with 20 lines per detector in different ranges.

For further evaluation, we performed a statistical analysis of the quality difference between the reference image and a reconstructed one. We compared reconstructions made with matrices generated with 20 lines per detector. The results are summurized in Table 3. The similarity of these results with the magnified images in Fig 10 proves that the system matrix generated in the range 0–90 degrees is sufficient to recontruct the images. The difference of MSE and PSNR in Table 3 between the first and two other lines could be due to the fact that the initial set of the data that corresponds to the range 0–180 degrees has been extended to the range 0–360 degrees in order to be used with the corresponding matrix in this range. An additional factor is the cumulative computational error [15] that causes small differences, also, in the ranges 0–90 and 0–180 degrees.

**Table 3:** Quantitative comparison of the images reconstructed with LSQR using matrices in different ranges.

Matrix range generation	MSE	PSNR
0–360 degrees	0.0820	58.9929
0–180 degrees	0.0680	59.8057
0–90 degrees	0.0658	59.9469

Finally, it should be mentioned that in the ranges 0–90 and 0–180 degrees, the images are reconstructed from 50 projections, which means that the radiation dose delivered to patients is reduced by a factor of 4.

## Conclusions

We analyzed the system matrix in the reconstruction process of CT images. The system matrix contains information on the acquisition geometry of the scanning process. The generation of the matrix elements affects the quality of the resulting image.

We demonstrated the capacity of the Siddon method to generate the system matrix with which good-quality images can be reconstructed from a limited number of projections. Moreover, the symmetry of the data structure allows the reduction of the system’s memory usage and of the reconstruction time.

We further demonstrated that, using the system matrix generated in the range 0–90 degrees, it is possible to reconstruct images from a limited number of projections, which allows the reduction of the radiation dose delivered to patients.

The system matrix can be pre-computed, saved, and reused, thus reducing the time of the entire reconstruction process. The obtained results could have practical application in portable scanner devices.

In the future, we will continue to use the symmetric block structure of the matrix to further reduce the range of the initial matrix to 0–45 degrees. We also plan to apply the Siddon method to generate the elements of the system matrix on fly during the reconstruction process, thus minimizing the memory usage of the system.

## Supporting Information

S1 Dataset Projection data set1 (50 views). (TXT)

S2 Dataset Projection data set2 (100 views). (TXT)
